# A longitudinal multidimensional rehabilitation program for patients undergoing allogeneic blood and marrow transplantation (CaRE-4-alloBMT): Protocol for a phase II feasibility pilot randomized controlled trial

**DOI:** 10.1371/journal.pone.0285420

**Published:** 2023-05-16

**Authors:** Samantha Tam, Rajat Kumar, Paty Lopez, Jonas Mattsson, Shabbir Alibhai, Eshetu G. Atenafu, Lori J. Bernstein, Eugene Chang, Susan Clarke, David Langelier, Jeffrey Lipton, Samantha Mayo, Tina Papadakos, Jennifer Michelle Jones

**Affiliations:** 1 Institute of Medical Science, University of Toronto, Toronto, Ontario, Canada; 2 Department of Supportive Care, Princess Margaret Cancer Centre, University Health Network, Toronto, Ontario, Canada; 3 Hans Messner Allogeneic Transplant Program, Princess Margaret Cancer Centre, University Health Network, University of Toronto, Toronto, Ontario, Canada; 4 General Internal Medicine, University Health Network, Toronto, Ontario, Canada; 5 Department of Medicine, University of Toronto, Toronto, Ontario, Canada; 6 Department of Biostatistics, Princess Margaret Cancer Centre, University of Toronto, Toronto, Ontario, Canada; 7 Department of Psychiatry, University of Toronto, Toronto, Ontario, Canada; 8 Faculty of Nursing, Princess Margaret Cancer Centre, University Health Network, University of Toronto, Toronto, Ontario, Canada; 9 Cancer Education, Princess Margaret Cancer Centre, Toronto, Ontario, Canada; 10 Patient Education, Ontario Health, Cancer Care Ontario, Toronto, Ontario, Canada; University Hospital Cologne: Uniklinik Koln, GERMANY

## Abstract

**Background:**

Allogeneic blood and marrow transplantation (alloBMT) is a curative treatment for blood cancers associated with various treatment-related adverse events and morbidities. Current rehabilitation programs are limited for patients undergoing alloBMT and research is urgently needed to test the acceptability and effectiveness of these programs. In response, we developed a 6-month multidimensional longitudinal rehabilitation program that spans from pre transplant to 3 months post transplant discharge (CaRE-4-alloBMT).

**Methods:**

This study is a phase II randomized controlled trial (RCT) conducted at the Princess Margaret Cancer Centre in patients undergoing alloBMT. A total of 80 patients stratified by frailty score will be randomized to receive usual care (n = 40) or CaRE-4-alloBMT plus usual care (n = 40). The CaRE-4-alloBMT program includes individualized exercise prescriptions, access to online education through a dedicated self-management platform, wearable technology for remote monitoring, and remote tailored clinical support. Feasibility will be assessed by examining the recruitment and retention rates and adherence to the intervention. Safety events will be monitored. Acceptability of the intervention will be assessed through qualitative interviews. Secondary clinical outcomes will be collected through questionnaires and physiological assessments at baseline (T0, 2–6 weeks pre-transplant), transplant hospital admission (T1), hospital discharge (T2), and 3 months post-discharge (T3).

**Conclusion:**

This pilot RCT study will determine the feasibility and acceptability of the intervention and study design and will inform full-scale RCT planning.

## Introduction

Allogeneic blood and marrow transplantation (alloBMT) is a curative treatment for hematologic cancers and its use has increased rapidly over the past decade [1, 2]. While effective, alloBMT is associated with very high disease burden including numerous treatment-related physical and psychosocial side effects, reduced physical functioning, and worsening nutritional status, which are associated with increased risk of complications, early and late treatment-related mortality, and impaired quality of life (QoL) [3–9]. As a result, there have been calls for proactive approaches to address the adverse effects associated with alloBMT with the goal to minimize dysfunction, maximize well-being and QoL, and reduce treatment-related mortality [10–13].

Cancer rehabilitation focuses on the prevention and treatment of cancer-related sequelae while optimizing functional independence and QoL [14–18]. Currently, longitudinal rehabilitation programs are not typically available in transplant centres [19] and research on rehabilitation interventions in alloBMT has been limited [20]. Encouragingly, the studies conducted to date appear promising and report improvements in physical functioning and QoL [20, 21] but limitations have been highlighted and include heterogeneity of study design and outcomes, small sample sizes, poor adherence, and high attrition rates due to the clinical complexity of this population [22–25]. Further, the majority of these interventions have taken a mono-dimensional approach (i.e. exercise), focused on one specific time point within continuum of care (i.e. pre-transplant [21, 24, 26], post-transplant [25, 27], or during hospital admission [28, 29]) and have been delivered exclusively in person [20, 21]. Moving forward, research is urgently needed to test the impact of longitudinal (pre, peri and post transplant) multidimensional rehabilitation programs that provide holistic care to this complex patient population [22]. Further, research could help to understand the role of technology and e-Health in improving access to cancer rehabilitation and overcome some of the adherence and attrition issues experienced in this population. The delivery of virtual rehabilitation has been well established in other populations such as cardiac rehabilitation and has equivalent efficacy and safety compared to in-person delivery and superior adherence [30]. Virtual and telehealth-based cancer rehabilitation interventions have been shown to reduce disability for cancer patients [31, 32], though more research is needed [32].

In response, through a strong collaboration between the Cancer Rehabilitation and Survivorship (CRS) and Hans Messner Allogenic BMT Programs at the Princess Margaret Cancer Centre, we developed an innovative multidimensional rehabilitation intervention for patients undergoing alloBMT (CaRE-4-alloBMT) which spans from pre to post transplant and utilizes e-Health and wearable technology. In line with standard feasibility objectives [33], the primary objectives of this study are: 1) to assess the feasibility, acceptability, and safety of the CaRE-4-alloBMT program (intervention adherence and attrition, acceptability of the intervention, adverse events); and 2) to examine the feasibility of the study methodology (recruitment and retention, feasibility of data collection instruments and procedures). The secondary objective is to describe the preliminary effects of the CaRE-4-alloBMT on disability, physical functioning, QoL, mood, cognitive functioning and physiologic outcomes including cardio metabolic factors, upper and lower body strength, and aerobic functional capacity.

## Methods

### Study design

**Fig 1:**
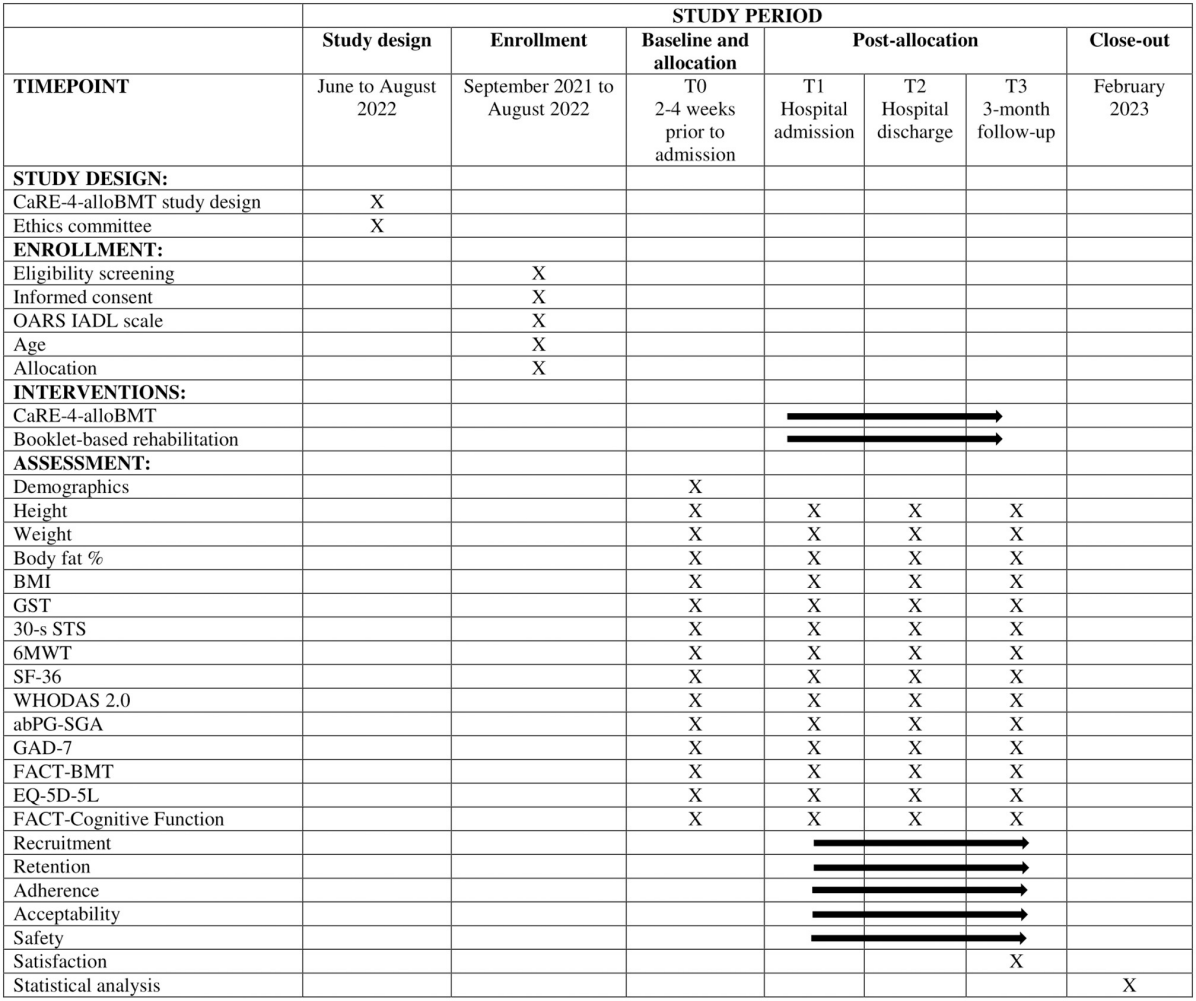
SPIRIT statement.

**Fig 2:**
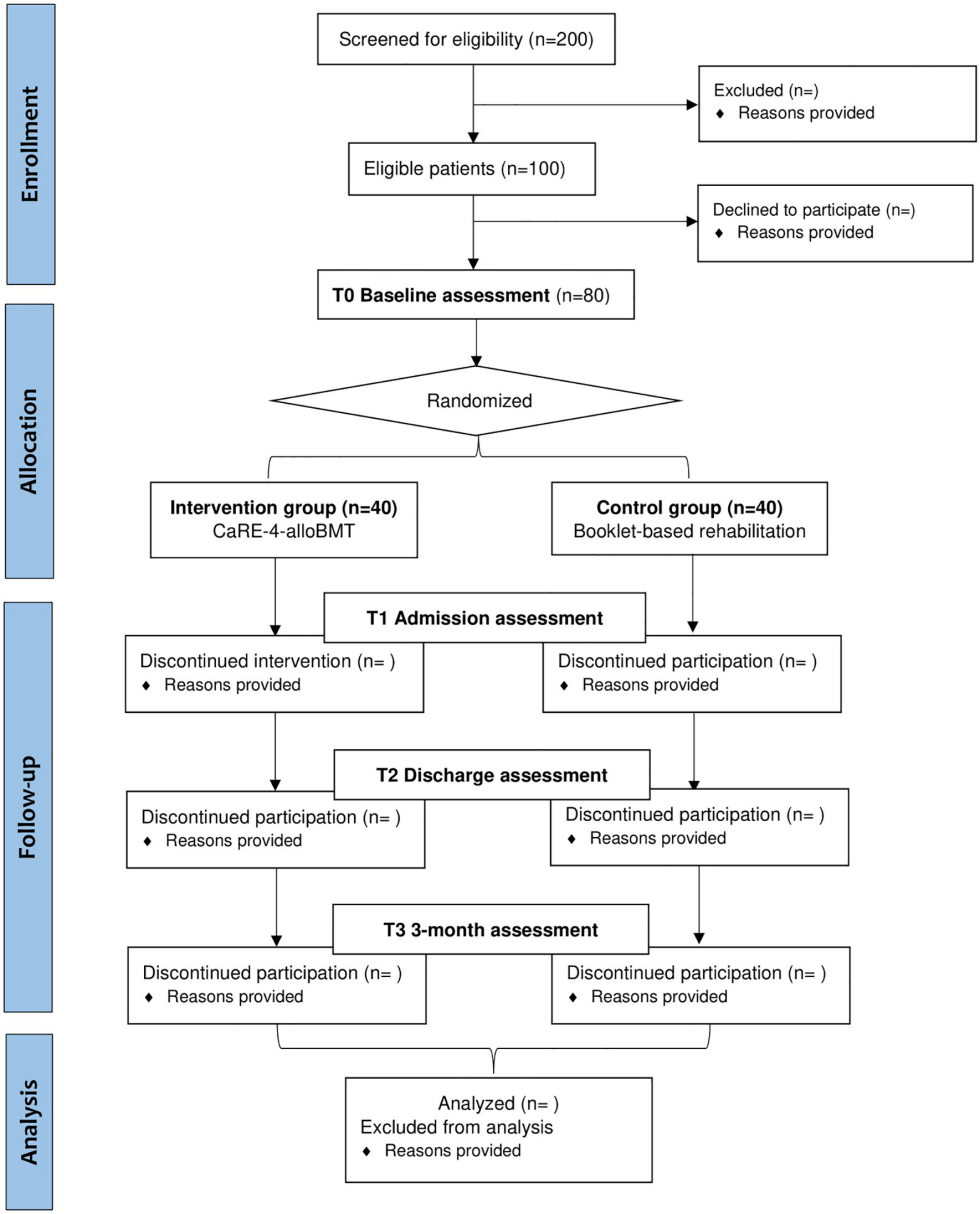
CONSORT participant flow diagram.

**Fig 3:**
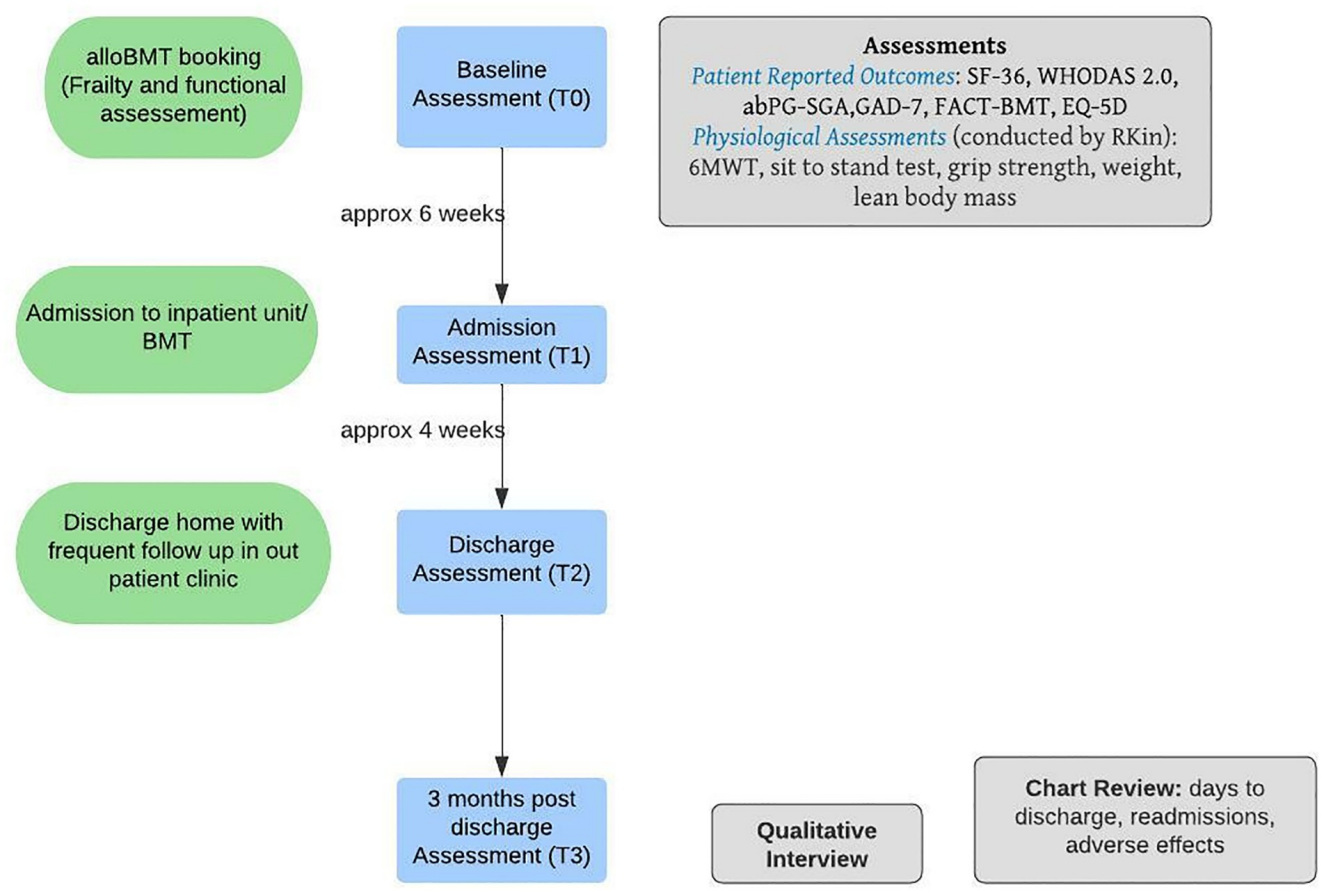
Study flowchart and timeline.

The proposed study is a single-centre Phase II RCT with patients scheduled to undergo alloBMT at the Hans Messner Allogenic BMT Program at the Princess Margaret Cancer Centre, Toronto, Canada. The study protocol is reported according to the Standard Protocol Items: Recommendations for Interventional Trials (SPIRIT) 2013 [33, 34] (see Fig 1). A SPIRIT checklist is included in S1 Checklist. A CONSORT [34, 35] participant flow diagram is shown in Fig 2. Following enrollment (2–6 week pre-transplant), participants will complete the baseline assessment (T0) and will then be randomly allocated to either receive usual care (UC) or usual care plus CaRE-4-alloBMT (INT). They will complete follow-up assessments at alloBMT admission (T1), discharge (T2) and 3 months post discharge (T3) (see study flow Fig 3).

This trial has been registered with clinical trials.gov (NCT04966156) and has been approved by the University Health Network Research Ethics Board (REB#21–5076).

#### Study population and recruitment

Eligible participants will be scheduled to undergo alloBMT at Princess Margaret Cancer Centre and will include people who are: 1) ≥ 18years of age; 2) receiving treatment for a hematologic cancer diagnosis; 3) completing pre and post transplant care at Princess Margaret Cancer Centre; 4) able to access on-line study material; and, 5) able to understand (spoken and written) English. Participants receiving a second transplant at the time of screening will be included given they meet the above criteria. Participants who do not meet the above criteria will be excluded from the study. In addition, patients who are scheduled to be admitted to the hospital for their alloBMT ≤ 2 weeks from the initial assessment booking will be excluded, as this will not allow enough time for the prehabilitation phase of the study. Further, those participating in any other clinical trials or intervention studies will also be excluded from this study.

Potential eligible participants will be identified from weekly-generated clinic lists by the study research assistant (RA) and transplant nurse coordinators during the initial pre-transplant consultation. The transplant nurse coordinators will first introduce the study to the patient by a study information letter which will be added to the information package that patients receive at this appointment. The RA will then speak to potential patients about the study and answer any questions they may have. Following the initial consult, the clinic staff will provide the RA with a list of patients who have consented to transplant. The RA will contact the patients by telephone or email to review the consent form. While in-person will be the primary method for obtaining consent from participants, we will also offer the option to provide consent virtually via Research Electronic Data Capture tools (REDCap^®^) [36]. Consenting participants will be scheduled for their baseline assessment. To optimize retention and prevent attrition, we will ensure frequent contact and monitoring with participants [37], and will use a collaborative- patient-centric approach including working individually with the patient, communication of the rationale for each phase of the study, allowing flexibility in data collection methods, and promoting shared accountability and responsibility in addressing the patients’ needs.

#### Randomization and blinding

A stratified randomization (Frail vs. Not Frail in the ratio of 7:13) list in 1:1 ratio to either INT or UC will be prepared before the T0 assessment. Once eligible participants consent to the study, random allocation will take place by a biostatistician and research co-ordinator. The randomization will be stratified by clinical frailty score due to its influence on alloBMT outcomes (Frailty score of 14 versus <14). Frailty will be assessed at alloBMT booking using the Older Americans Resources and Services instrumental activities of daily living (OARS IADL) [38]. The allocation sequence will be computer-generated block randomization and will be concealed from study staff at recruitment and baseline assessment. Given the nature of the intervention, it will not be possible to blind participants and study staff once group assignment is revealed. However, study staff conducting the physiological assessments will follow a structured standardised testing protocol [39].

#### Withdrawal and replacement of individual participants

Participants will be informed that they can withdraw from the study at any time without any consequences. Participants who withdraw from the study prior to randomization will be replaced. However, participants who withdraw after randomization will not be replaced. In both cases, they will be asked to share the reason for withdrawing from the study. The investigator may withdraw a participant from the study for urgent medical reasons or if the alloBMT is cancelled prior to baseline assessment.

### Interventions

#### CaRE-4-alloBMT

**Fig 4:**
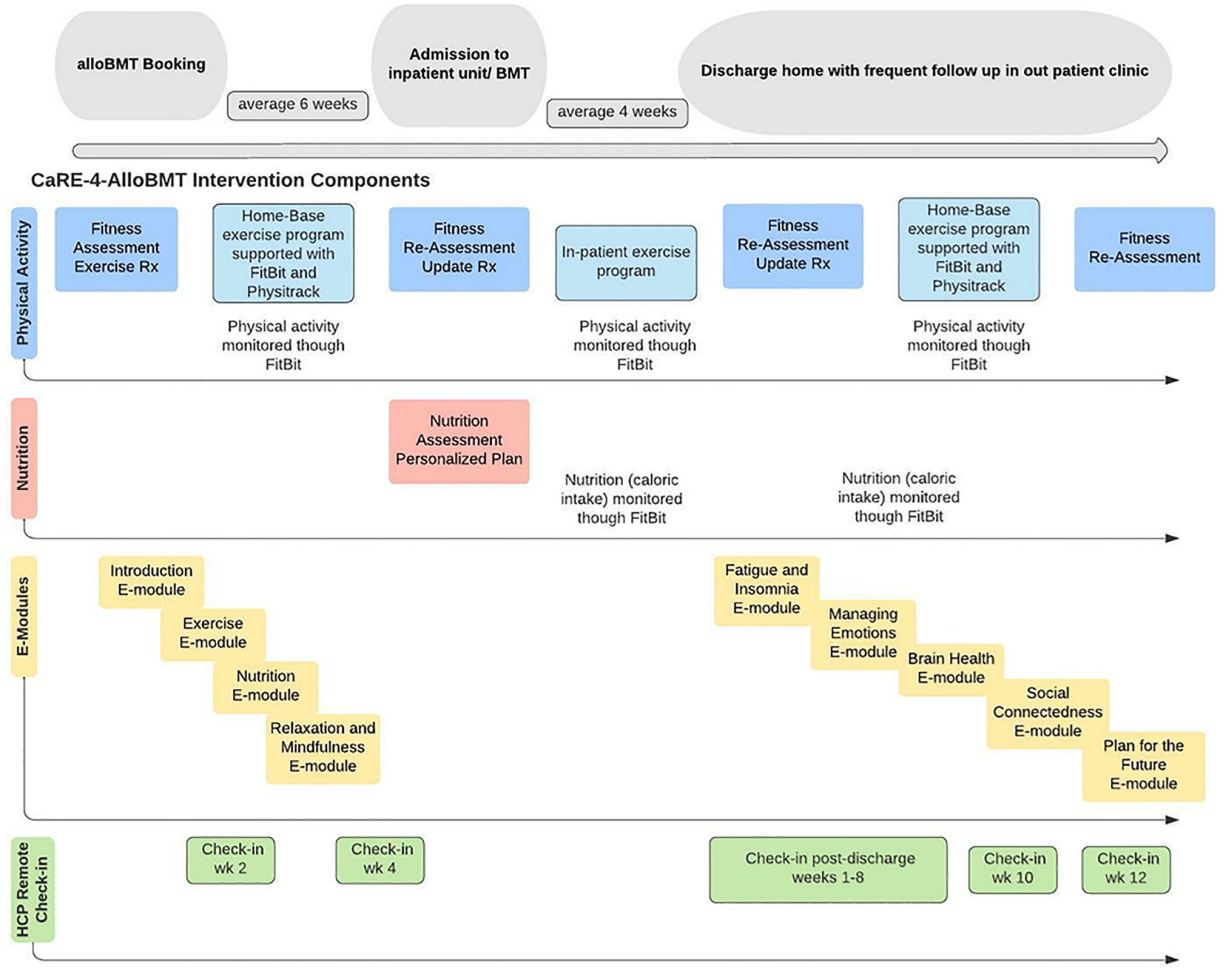
Timeline of the delivery of intervention components.

Patients randomized to the intervention (INT) arm will receive usual care plus the CaRE-4-alloBMT intervention. CaRE-4-alloBMT is a longitudinal 6-month rehabilitation program (peri to post transplant) adapted from an existing evidence-based and effective model developed by the CRS team [40, 41]. CaRE-4-alloBMT uses a person-centred strategy and a multidimensional approach targeting physical activity, nutrition, psychosocial distress and promoting self-management skills to manage common side effects. The program is was developed based on established behaviour change theory, including motivational interviewing (MI) [42], the theory of planned behaviour [43], self–efficacy [44], and the model of supportive accountability [45]. Further, there are a number of number of embedded behaviour change techniques, which are known to result in larger effects [46–50]. With the exception of the assessments, the program is virtual and utilizes current and emerging eHealth technologies in order to reduce barriers to accessing and providing cancer rehabilitation [40, 41, 51, 52]. Innovative components of CaRE-4-alloBMT include: 1) Progressive tailored exercise prescription that is supported with a mobile application (Physitrack^®^); 2) Remote monitoring with wearable technology (Fitbit^™^); 3) Self-management skill teaching through interactive eLearning modules; and 4) Scheduled remote check-ins and health coaching via video or telephone. Fig 4 presents the timeline in which intervention components will be delivered.

Individualized progressive exercise prescriptions: Based on the baseline physical fitness assessment, an individualized and progressive exercise prescription will be developed and monitored by an experienced registered kinesiologist (RKin). Each exercise program will contain cardiovascular and strength training as well as flexibility exercises [53] and will be adjusted to the needs of the patient and their reduced physical functioning during hospitalization and early days post transplant [54]. The program will aim to reach at least 90 minutes of moderate intensity aerobic exercise per week, resistance exercises 2–3 times per week focusing on major muscle groups, and flexibility stretching for major muscle groups 2–3 times per week [53]. The exercise program will be revised and modified based on the individual’s needs at the follow-up assessments or during the scheduled check-ins between assessments. The tailored exercise prescription will be supported by Physitrack^®^, which is an online application that allows customizable exercise prescriptions, videos of exercises, and tracking of exercise completion [40]. The participants will be provided with an orientation to the Physitrack^®^ application by the RKin. If the participant is unable to download or use Physitrack^®^, they will be provided with a detailed print out of their exercise program.

Remote monitoring: Fitbit^™^ devices will be used to monitor patients’ physical activity, caloric intake, and sleep for the duration of the program. The assigned study RKin will have access to Fitbit^™^ data in order to monitor these metrics. The Fitbit^™^ tracker is also known to promote behaviour change and allows for self-monitoring and feedback to the participant [40, 51]. Participants will be asked to wear the Fitbit^™^ for the duration of the study and to log their meals on their Fitbit^™^ account in order to monitor their nutrition and caloric intake. The RA will provide support to the participant as needed to set-up the device and access the Fitbit^™^ application.

Self-management skills teaching: Participants will be registered into an online learning platform and asked to complete a total of nine brief (15-25min) interactive eLearning modules (see Fig 4). The dedicated self-management platform including eLearning modules and goal setting tools were developed by the Princess Margaret Cancer Self-Management Research Centre in collaboration with the CRS and Hans Messner Allogenic BMT Program teams. The platform and modules include health literate design features and are informed by principles of adult learning and self-management theory [40]. Behaviour change techniques were embedded into the modules incluing interactive elements used to strengthen behaviour change goals. Participants are asked to first go through the welcome video: Introduction to CaRE-4-alloBMT. Participants are then asked to complete the first four eLearning modules: Getting Started; The Role of Exercise; Eat and Cook for Wellness; and Be Mindful before they are admitted for transplant. Following transplant, and after the patient has been discharged to home (3-month period), they are asked to complete the five remaining eLearning modules: Improve Fatigue and Sleep; Manage Your Emotions; Brain Health; Find Ways to Connect; and Plan for the Future.

Remote person-to-person clinical support: During pre/post discharge phases, patients will have scheduled remote check-ins and health coaching sessions with their RKin. The addition of a person-to-person component is an important component in the delivery of online behaviour change interventions [55] as it creates accountability, and provides opportunity for tailored feedback and social support [45]. Check-ins will be offered via MS Teams video or telephone and scheduled at weeks 2 and 4 after initial assessment (pre-alloBMT admission) and then weeks 1–8, 10 and 12 post-BMT discharge (see Fig 4). During these calls the RKin will review, adapt, and progress the exercise prescription as needed, guide the participant to reflect upon what has happened since their last check-in, discuss and develop goals for the follow week, and to identify potential barriers and solutions in achieving their goals. The study RKins have been trained in MI by a certified Motivational Interviewing Network Trainer and will incorporate the assessment and promotion of intrinsic motivation, development of self-efficacy, and will use a collaborative problem solving approach [42]. MI elicits and strengthens motivation for change by identifying and addressing ambivalence [42] and it has been shown to be effective in increasing physical activity in populations with chronic conditions including cancer [40–42, 51, 56].

Individualized nutrition plans: As part of standard of care, individualized nutrition plans based on nutritional status will be delivered by registered dieticians (RD) from the alloBMT team. Those in the INT group will receive additional nutritional education through the eLearning modules. In addition, caloric intake will be monitored using the Fitbit^™^ tracker. Patients will be taught by the RA how to track their nutritional intake on their Fitbit^™^ device, and this data will be monitored by the RKin. Patients who fall below 50% of their caloric intake goals (based on RD completed intake assessment) will be flagged to the RD for follow up.

#### Usual care

Participants randomized to the usual care group will receive standard best practice care that is provided to all alloBMT patients. Access to in-patient and out-patient rehabilitation will be provided as determined by the alloBMT team and will be tracked and documented by the study RA. As part of standard of care, patients are provided with links to self-directed online education resources. This includes an education booklet on physiotherapy for alloBMT which outlines the importance of physiotherapy, the benefits of exercise which includes cardiovascular and resistance exercise targets, and suggests simple exercises that can be completed in the hospital including strengthening exercises, riding the stationary bicycle, and walking. In addition, all patients receive a nutrition assessment at admission and caloric intake goals are set by the alloBMT dietitian. All usual group participants will complete the physiological assessments at all four time points and will be provided with the results. UC participants will not be given any fitness instruction by the study RKin.

### Outcomes

#### Primary outcomes

Feasibility of the intervention and methods assessed are summarized in Fig 1 and include the following: 1) Recruitment and eligibility rates; 2) Retention and attrition; 3) Intervention adherence; 4) Treatment implementation and fidelity. A Consolidated Standards of Reporting Trials diagram adapted for pilot feasibility studies (see Fig 2) will be used to track participant flow throughout the study [34].

To evaluate acceptability and inform program modification, we will conduct semi-structured qualitative video interviews via telephone or MS Teams with a sub-sample of approximately 10–15 INT participants following the final T3 assessment. The purpose of the qualitative interviews is to gain feedback on program and research acceptability and impact [57]. All interviews will be conducted using a semi-structured interview guide (see S2 File) and will be digitally recorded and transcribed verbatim. An interpretive descriptive qualitative methodology will be used [57–61].

Safety of the intervention will be assessed throughout the study and any adverse events resulting from the intervention will be scored on the CTCAE version 5.0 [62] and documented during weekly health coaching calls with the RKin. Assessment tests and all exercises will be stopped at any time if any pain or discomfort is experienced.

Fig 1 outlines the schedule of outcomes.

1. Recruitment rate will be assessed based on CONSORT criteria [35] through a screening log that tracks data collected of all screened patients. Eligibility screening will be performed to identify eligible consented and eligible non recruited individuals with non recruitment reasons documented. Rates will be calculated using the number of participants who consent to participate compared to the number of eligible patients.
2. Retention rates at each study time point will be monitored with reasons for drop out documented. Based on available literature for this patient population, a retention rate of at least 70% will be used to determine good retention for the study [27–29, 63–65]. Retention rates will be calculated as the proportion of participants that attend each assessment time point (T0, T1, T2, and T3). We will also examine rates of complete and missing data from questionnaires and physical assesments.
3. Adherence to the intervention will be assessed through health coaching call attendance, Fitbit^™^ and Physitrack® use, and eLearning module completion.
4. Treatment implementation and fidelity will be assessed throughout the study in order to ensure consistency for all participants across the duration of treatment [66]. This will include delivery of health coaching calls to intervention participants on MS teams by the RKin at weeks 2,4 (pre) and 1–8,10,12 (post-discharge). Pre-developed motivational health coaching interview approach will be used by the RKin. Attendance will be documented and analyzed, including any modifications to health coaching call sessions (eg. Added calls, adjusting the duration of call sessions to suit patient needs).

#### Secondary outcomes

Patient reported outcomes measures and physiologic assessments will be completed at baseline (T0), alloBMT hospital admission (T1), hospital discharge (T2), and 3-months post-discharge (T3) (see Fig 3).

Patient reported outcomes will be completed online via RedCap^™^ or on paper at each time point. The specific measures included are detailed below.

Health Status
  - The 36-Item Short Form Survey (SF-36) is a widely used measure of health status and the impact of clinical and social interventions [67]. The measure contains 36 items and summary scores can be calculated for a physical component and a mental health component [68]. In the alloBMT population, the Physical Component Summary scores are predictive of transplant-related and overall mortality [26].
Disability
  - The 12-item World Health Organization’s Disability Assessment Schedule 2.0 (WHO-DAS 2.0) was developed by the World Health Organization to measure disability and is aligned closely to the International Classification of Functioning, Disability and Health [69, 70]. Respondents rate their difficulty in engaging in particular activities on a scale from “none” (no difficulty) to “extreme or cannot do” on six domains of functioning. Scores range from 0 to 48, with higher scores indicating higher levels of disability or loss of function.
Nutritional Status
  - The Abridged Scored Patient-Generated subjective Global Assessment (abPG-SGA) is a four-part questionnaire that assesses patients’ weight history, food intake, appetite, and performance status and considered an acceptable measure to identify malnutrition in cancer patients [71].
Anxiety
  - The Generalized Anxiety Disorder Questionnaire (GAD-7) is a seven-item measure to assess the core symptoms of generalized anxiety disorder [72, 73]. Items are scored on a 4-point scale and scores can range from 0 to 21, with higher scores indicating higher anxiety.
Quality of Life
  - The Functional Assessment of Cancer Therapy–Bone Marrow Transplantation (FACT-BMT), is a multi-dimensional quality of life measure that has been developed for BMT patients [74]. It consists of the 27-item FACT-General and the 23-item Bone Marrow Transplantation Subscale.
  - The EQ-5D-5L is used in oncology to generate health-related QoL weights and corresponding health states [75].
Cognitive Function
  - The FACT-Cognitive Function Questionnaire (3.0) is a 37-item measure to assess perceived cognitive function in cancer populations. It have been widely used across clinical settings including the BMT population [76, 77].

Physiological assessments [78] will be conducted in-person on all participants by a trained RKin and will include the following:

Cardiovascular health screening
  - Including resting heart rate, blood pressure, SPO_2_
Body composition
  - Height, weight, body mass index (BMI) and body fat %.
Strength
  - Upper-extremity strength will be measured via handgrip dynamometry or grip strength test (GST) [79]. This test will be performed in a standing position with the upper limbs held straight and the wrist kept at a neutral position. Both right and left hands will be given two tries, and the highest readings of each will be added and used in analysis.
  - Lower limb muscle strength and endurance will be measured by a 30-second sit to stand test (30-s STS) [80]. This test will be performed with the participant in a sitting position, arms crossed over the chest, and hips and knees bent at 90°. The participant will stand up and sit down repeatedly, and the number of repetitions completed in 30 seconds will be recorded.
Aerobic functional capacity
  - Aerobic functional capacity will be measured using a 6-minute walk test (6MWT) [81]. This test has been shown to be safe, easy to administer, well tolerated and is a good measure of activities of daily living [82]. In a straight hallway of 30 or 50m, patients will be asked to walk from one end to the other. At admission and discharge assessments, the 6MWT will be modified to walking around the in-patient units for ease of access. The total distance will be recorded for analysis. Post-assessment cardiovascular health screening will be conducted to ensure the safety of the participant.

#### Sample size

There remains a lack of consensus regarding appropriate sample size for feasibility studies [83]. However, using a simulation of a range of sample sizes and values of standard deviation for precision of estimate with α = 0.05 and power at 80%, the elbow point of the curves is at 35–40. Based on this, a sample size of 80 participants (40 participants in each arm) will be included in the study. This is considered large enough to examine the feasibility of the study [84, 85]. Based on program volumes (~200 alloBMT/year), we plan to recruit our sample over a 10–12-month period.

### Data management

A detailed database will be developed to track each participant’s progress and will include notes on any clinical or study deviations. All study data will be stored in encrypted files on secure servers at the University Health Network and outcome data will be stored on REDCap^®^ [36] which is a secure web-based application for electronic data. Data files will be restricted to study investigators and authorised study personnel. Data quality and integrity will be assured using data audits, access control, and monitoring and cleaning data.

#### Primary outcomes

Study feasibility will be evaluated using descriptive statistics and will include: 1) the number and proportion of patients who are eligible (and reasons for ineligibility); 2) the number who provide consent (and reasons for declining); 3) attrition rates at each time point (and reasons for drop out); and 4) capture of outcome data at each time point and missing items. Feasibility of the intervention will be assessed by examining: 1) Completion of the eLearning modules; 2) Use of the Fitbit^™^ (from T0-T3); and 3) Remote clinical check in call attendance and any added calls.

Acceptability will be measured through the interview data and a thematic analysis will be conducted [86]. Analyses will be primarily deductive and codes/categories will be pre-determined to align with the main objectives (see S2 File) [58, 60, 61, 86]. Following the initial coding, inductive coding will be conducted to identify any additional codes. Themes will be created through examination of the final codes/categories and their relationships.

Any safety event related to the program will be reported using Common Terminology Criteria for Adverse Events v5.0 [62].

#### Secondary outcomes

Variability of the main and interaction effects will be examined using separate repeated measures ANCOVA models and corrections for multiple comparisons will be applied. Hedges’ g and associated confidence intervals [87] will be calculated in order to estimate of the effect size within and between groups [88]. Missing data will be evaluated on a case-by-case basis and drop-outs will be excluded. Data will be analyzed when all recruitment and data collection has been completed.

#### Interpretation of results

We will define our intervention as feasible to test in a larger, multi-center RCT if there is reasonable recruitment (≥40% of all eligible participants) [25, 28, 29, 89, 90], retention (70%) [25, 27, 28, 63, 64, 90] and at least 80% capture of outcomes, and reasonable adherence to the intervention components (70%) [25, 63–65, 89, 90]. In addition, success will be measured by high levels of treatment acceptability based on qualitative interview data. Safety of the intervention will be confirmed if no serious adverse events (defined as anything above a Grade 2 of the CTCAE v5.0) related to participation in the intervention occur. If any one of those criteria are not met, we will make appropriate modifications to the protocol prior to a larger RCT. Interpretation of the effect size and mean difference scores will be based on a minimally important clinical difference of 5 points (or 0.5 SD) between the experimental groups on the SF-36 Physical Function Summary score at the T3 assessment [26, 91].

## Discussion

Embedding multidimensional longitudinal rehabilitation programs using innovative delivery strategies as a standard part of treatment for individuals undergoing alloBMT has potential to mediate the significant adverse effects, improve disability and physical functioning and QoL, and reduce burden on the healthcare system [13, 63, 64, 92–96]. However, longitudinal rehabilitation programs are not typically available in transplant centres [19, 20]. We attempt to address this gap with this study. The CaRE-4-alloBMT study uses a person-centered strategy and multidimensional approach to target physical activity, nutrition, and to promote self-management skills to support improved function [40, 41]. The primary objective of this study is to assess the feasibility, acceptability and safety of the CaRE-4-alloBMT program. The results from this pilot will provide foundational information that will inform the development of a Phase III RCT.

### Strengths and limitations

The study and intervention design have a number of strengths. As described, the CaRE-4-alloBMT program is based on pre-existing clinical and research experience, established theory, and contains embedded behaviour change techniques. This is a collaborative project that involves both the alloBMT and rehab clinical teams which, will help to support buy in and recruitment for the study [97]. The use of eHealth technology including eLearning modules, wearable technology and the exercise application as well as tailored person-to-person support and supervision provides the convenience of completing the program remotely and helps to promote effectiveness and adherence [55]. Finally, the study has been designed following the SPIRIT 2013 Statement (Standard Protocol Items: Recommendations for Interventional Trials) and careful attention has been taken to optimize methodological quality.

We also recognize that this study design has limitations. To begin, this study is being conducted in a single comprehensive tertiary care centre, located in an urban setting, which may limit generalizability. Further, due to the nature of the intervention, funding and staffing, it will not be possible to conduct this as a double-blind study. While assessors will be trained to follow a standardized detailed protocol [39], biases cannot be ruled out and will need to be accounted for. All anticipated and unanticipated challenges will be noted and solutions will be documented to refine the program and study design in order to inform full-scale RCT planning.

## Supporting information

S1 Checklist SPIRIT 2013 checklist. (DOC)

S1 File (DOCX)

S2 File CaRE-4-alloBMT qualitative interview guide. (TIFF)
