# Supplementary material for: A longitudinal multidimensional rehabilitation program for patients undergoing allogeneic blood and marrow transplantation (CaRE-4-alloBMT): Protocol for a phase II feasibility pilot randomized controlled trial
Source: PLoS One. 2023 May 16;18(5):e0285420. doi: 10.1371/journal.pone.0285420 (PMC10187908; doi:10.1371/journal.pone.0285420)
Supplement: S1 File — (DOCX) [file pone.0285420.s002.docx]

**A LONGITUDINAL MULTIDIMENSIONAL CANCER REHABILITATION PROGRAM FOR PATIENTS UNDERGOING ALLOGENEIC BLOOD AND MARROW TRANSPLANTATION**

**PRINCIPAL INVESTIGATORS:**

1. Jennifer M Jones, PhD

Senior Scientist and Director, Cancer Rehabilitation and Survivorship Program (CRS)

2. Rajat Kumar, MD, MSc, FRCPC

Head, Malignant Hematology Service and Hematologist, Allogeneic BMT program

**CO-INVESTIGATORS:**

1) Jonas Mattsson, Gloria and Seymour Epstein Chair in Cell Therapy and Transplantation and

Director, Hans Messner Allogeneic Transplant Program

2) Jeffrey Lipton, Oncologist and Clinical Investigator

3) Lori Bernstein, CRS Scientist and Neuropsychologist

4) David Langelier, CRS Physiatrist and Scientist

5) Eugene Chang, CRS Physiatrist and Medical Lead

6) Samantha Mayo, RBC Financial Group Chair in Oncology Nursing Research

7) Wanrudee Isaranuwatchai, Director, Centre for Excellence in Economic Analysis Research

**COLLABORATORS:**

1) Aleksandra Chafranskaia, CRS Clinical Manager

2) Susan Clarke, Nurse Manager, Inpatient Allogeneic BMT Units & Allogeneic Pre- Transplant

3) Stephanie Gladman, CRS Registered Dietitian

4) Michele Lynn Heffering, AlloBMT Nurse Coordinator

5) Paty Lopez, CRS Kinesiologist

6) Calvin Mach, AlloBMT Registered Dietitian

7) Manjula Maganti, UHN Biostatistician

8) Stephanie Phan, CRS Occupational Therapist

9) Leeann Wilson, AlloBMT Nurse Coordinator

10) Anna Wong, CRS Physiotherapist

11) Connie Ziembicki, AlloBMT Registered Dietitian

**1.0 BACKGROUND:** Allogeneic blood and marrow transplantation (alloBMT) is a curative treatment for many hematologic cancers and its use has increased rapidly over the past decade. While effective, alloBMT is associated with numerous treatment-related physical and psychosocial side effects, reduced physical functioning, and worsening nutritional status which are associated with increased risk of complications (i.e. infections, drug toxicity, weight loss, graft failure & graft‐versus‐host disease), significant early and late treatment related mortality, and can profoundly affect quality of life (QoL).

As a result, ***there have been calls for increased attention to the management of adverse effects associated with alloBMT with the goal to minimize dysfunction, maximize well-being and QoL, and reduce treatment-related mortality*.**

Cancer rehabilitation is now considered an essential component of cancer care and focuses on

prevention and treatment of the adverse effects of cancer and treatment and to optimize functional status and QoL. Embedding multidimensional longitudinal (pre to post transplant) rehabilitation programs that focus on exercise, nutrition and teaching self-management skills as a standard part of treatment for individuals undergoing alloBMT has potential to mediate the significant adverse effects, improve survival, and reduce burden on the healthcare system. While patients typically receive some in-patient rehabilitation, this is a small part of the alloBMT pathway and services are quite limited. Currently, transplantation centers across North America generally do not offer longitudinal rehabilitation programs and research is urgently needed to test the acceptability and effectiveness of these programs using innovative delivery strategies that have the potential for future scalability and to

understand the associated costs. ***In response, through a strong collaboration between the PM Cancer Rehabilitation and Survivorship (CRS) and alloBMT teams, we developed an innovative multicomponent rehabilitation intervention for patients undergoing alloBMT (CaRE-4-alloBMT).***

**2.0 PROGRAM DESCRIPTION (*see Appendix 1*):** CaRE-4-alloBMT is a longitudinal 6-month rehabilitation program (peri to post transplant) adapted from an existing evidence-based and effective model developed by the CRS team (CaRE@ELLICSR and CaRE@Home). The program is informed by established behaviour change theory and harnesses current and emerging eHealth technologies to reduce barriers to accessing and providing cancer rehabilitation. CaRE-4-alloBMT uses a person- centred strategy and a multidimensional approach targeting physical activity, nutrition, psychosocial distress and promoting self-management skills. Innovative components of CaRE-4-alloBMT include:

**1) *Individualized progressive exercise prescriptions*** developed and monitored by CRS registered kinesiologists and supported with a web/mobile application (Physitrack) that allows customizable exercise prescriptions, tracking of exercise completion, and video tutorials. The initial personalized exercise program will be given to a participant following the initial assessment. A program will be created based on the specific needs and physical condition of each participant. The participant will receive the detailed exercise program via Physitrack (includes exercise description and videos of each exercise). Each exercise program will contain cardiovascular and strength training as well as stretching exercises. The exercise program will be revised and progressions will be added based on the individual’s needs after the follow-up assessments or during the scheduled check-ins between assessments. Check-ins are scheduled as follows:

- - At 2 and 4 weeks after initial assessment
  - 8 weekly check-ins following discharge
  - At 10 and 12 weeks following discharge

The participant can contact the Kinesiologist by e-mail at any point during the program.

**2) *Individualized nutrition plans and stepped stratified* care** (education, counselling, intervention) based on nutritional status and delivered by registered dietitians (alloBMT and CRS). As part of standard of care, all patients receive a nutrition assessment and their caloric intake goals are set. Those in the INT arm (CaRE-4-alloBMT plus usual care group) will receive additional nutritional education through the e-modules and monitoring of caloric intake through the Fitbit. They will also have access to meal preparation and recipes through ellicsrkitche.ca. Patients can track their nutritional intake on their Fitbit device and this data will be monitored weekly by the RKin. Patients who falls below 50% of their goals (based on intake assessment) will flagged to the RD for follow up.

**3) *On-line e-modules*** (developed in collaboration with PM Oncology Education) provide interactive education to promote self-management skills on crucial topics (i.e. stress management, nutrition, managing common symptoms). There will be 9 online e-modules:

- - Introduction: Getting started
  - Eat and Cook for Wellness
  - Exercise
  - Manage your emotions
  - Reduce Fatigue
  - Being Mindful
  - Boost your Brain Health
  - Stay Connected
  - Planning for the Future

Participants will be asked to complete all 9 modules. Each module should take between 20-30 minutes to complete. Participants can go through these modules at their own pace. Patients will receive a link for the online modules. Patients will be able to access the module by entering their e-mail address and creating their own unique password.

**4) *Remote monitoring using*** FitbitTM devices to monitor patients physical activity, caloric intake, and sleep for duration of the program. CRS and alloBMT clinicians will have access to real-time Fitbit data through our clinical dashboard (developed in collaboration with TECHNA). The Fitbit is also known to promote behaviours change and allows for self-monitoring and feedback. Participants will be asked to wear the Fitbit for the duration of the study. Participants will only need to add their meals to monitor their nutrition. No other action is required from the participants once the account has been created. This will replace the paper based food diary they must keep (standard of care). The account for the participant will be created by the RA or RKin and the participant will then be able to access the app via an email link. The RA or RKin can assist/provide support to the participant as needed to access the app. If the participant is unable to download or use the app a detailed print out of their exercise program will be provided.

The following information will be required to create an account:

- - First name
  - Last name (participant study number will be entered in this field
  - Year of birth
  - Email address

Both the participant and Kinesiology team will have access to this information but the account information is not visible to third parties. Physitrack provides links to the privacy policy and terms of use/agreement and is available for the participant to review as needed.

**5) *Remote clinical support:*** Pre/Post discharge, patients will have scheduled (PHS) remote check-ins and health coaching sessions with a member of the CRS team (phone or MS Teams video). These occur in week 2,4 (pre) and 1-8,10,12 (post-discharge).
*Frequency of check-ins and what will be discussed:* Check-ins are by telephone or MSteams video and scheduled as follows:

- - At 2 and 4 weeks after initial assessment (pre-BMT admission)
  - Weeks 1-8, 10 and 12 post-discharge (post-BMT dischange)
- Check-ins discussion using motivational interviewing techniques and focus on
  - Adherence to exercise program
  - Changes to exercise program based on participant’s needs
  - Module completion
  - Answering participants questions

The program strongly aligns with the SUNDAE framework (***see Appendix 1***).

**3.0 OBJECTIVES AND METHODS:**

**Objectives:** i) To test the feasibility and safety of CaRE-4-alloBMT plus standard best practice cancer

care compared to standard best practice cancer care alone; ii) To assess the preliminary efficacy of CaRE-4-alloBMT on *physical function*, *disability*, *nutritional status, distress, QoL, healthcare utilization, and survival* and estimate *program return on investment.*

**Methods (*see Appendix 2*):** We will conduct a Phase II feasibility RCT in patients undergoing alloBMT at Princess Margaret Cancer Centre. Patients will be randomized to receive usual care (n=40) or CaRE-4-alloBMT plus usual care (n=40). **Procedure:** Potential participants will be identified from weekly-generated clinic lists. Eligible participants will be identified by transplant nurse coordinators and oncologists during the initial pre-transplant consultation. Nurse coordinators/oncologist will introduce study to the patient. The study information letter will be added to the information package that patients receive at this appointment, if patient agrees to receive letter. The nurse coordinators/oncologist will provide the research assistant (RA) with a list of patients that agreed to be approached for this study.. The consent process will be opt in, and written informed consent will be obtained by the RA. After patients consent for transplant, the clinic staff will provide the study RA with a list of patients who have consented to transplant and have consented to be approached for study purposes. The RA will approach eligible patients at one of their usual care medical appointments to review consent form and answer any questions they may have regarding the study. If the patient agrees to participate and has no further questions, their consent will be obtained and a signed copy of the consent will be given to them. The RA will email patients a link to the REDCap questionnaire package a week prior to baseline assessment (T0); if preferred, patients can choose to complete a paper-based questionnaire at T0 instead. At baseline assessment (4-6 weeks pre-transplant), participants will undergo a 60-minute physical assessment. Following the assessment, patients will be randomized (1:1 stratified by age and clinical frailty score at pre-transplant consultation). Follow-up assessments will be conducted at: transplant hospital admission (T1), hospital discharge (T2), and 3-months post-discharge (T3). A week prior to each assessment, RA will email participants the link to the online questionnaire package. We will use REDCap, which is the preferred system for online questionnaires at UHN. If the participants prefer, they can complete a printed version of the questionnaire package on the day of the assessment instead of completing it online. Participants in the CaRE-4-alloBMT plus usual care group will also be asked to complete a Patient Satisfaction Survey at T3.

**While in-person will be the primary method for obtaining consent from participants, we recognize that not all participants are able to attend their appointments due to the effects of their illness and treatments. As such, we will also offer the option to provide consent virtually via REDCap.**

**Participant eligibility:** *Inclusion criteria*: 1) 18 years of age or older 2) Must have received a hematologic cancer diagnosis; 3) Awaiting alloBMT; 4) able to communicate in English; 5) able to access online study material (Physitrack and education e-modules). *Exclusion criteria:* 1) Not meeting the above criteria*.* **Outcomes: *Primary outcome:*** Feasibility and safety will be assessed and interpreted using quantitative and qualitative data and include *demand/uptake of the intervention, retention and adherence, and acceptability* in line with guidelines for feasibility studies. *Adverse events* that are a result of participation in the intervention will be documented. ***Secondary clinical outcomes:*** *physical function* (SF-36, 6MWT, Sit-to-stand and grip strength), *disability* (WHODAS 2.0), *nutritional status* (caloric intake, BMI, PG-SGA), *distress* (GAD-7), *QoL* (FACT-BMT, EQ5D), *healthcare utilization* (days in hospital, readmission), and *overall survival*. In addition to these patient related outcomes measures, demographic data will also be collected at baseline (only). These include: age, cancer diagnosis, gender, marital status, language, ethnicity, education, employment status, socioeconomic status, living arrangement, participation in other clinical/research studies, comfort level with English, exercise routine prior to and after cancer diagnosis and treatment as well as current exercise/activity. *Economic analysis:* compare the cost and effect between groups showing the intervention’s value for money from the payer perspective. **Analyses:** Quantitative feasibility outcomes will be summarized by descriptive statistics. Interview scripts (acceptability) will undergo inductive and deductive analyses and the constant comparative method.

For exploratory efficacy outcomes that are either continuous or categorical in nature, repeated measures analysis will be conducted by using Linear mixed models or GEE. **Sample size and timeline:** Based

on a simulation of a range of sample sizes and values of SD for precision of estimate (α=0.05 and power at 80%); 35-40 is at the elbow point of the curves. Therefore, sample size is 80 participants (40 per arm). This is in line with expectations for a pilot feasibility study. Based on program volumes (~200 alloBMT/year), we plan to recruit our sample over a 10-month period. Study set up has already begun (REB underway). The proposed timeline allows for 3 month follow-up and data analyses.

**4.0 TEAM:** Team members are researchers and clinicians and bring significant expertise related to

work proposed. **Co-Principal Investigators**: Dr. Jennifer Jones is a Senior Scientist and the Director of the CRS Program. She has a strong record of research in cancer rehabilitation and survivorship and in the development, evaluation and implementation of cancer survivorship and rehabilitation programs

and interventions. Dr. Rajat Kumar is Head of the Malignant Hematology Service and a Clinician Investigator specializing in the alloBMT site with a strong background in clinical trials. Our **study co- investigators** are clinician-investigators with expertise in oncology/BMT (Lipton, Mattsson), rehabilitation (Langelier, Chang), survivorship (Bernstein, Mayo), health economics (Isaranuwatchai). In addition, we have a large **team of collaborators** with expertise in nutrition (Gladman, Mach, Ziembicki), exercise (Lopez), nursing (Clarke, Heffering, Wilson), physiotherapy (Chafranskaia, Wong), occupational therapy (Phan), patient education (Papadakos) and biostatistics (Maganti).

**5.0 DELIVERABLES: *Data from this trial will be an important first step in the development of an internationally recognized collaborative program with high potential for impact.*** The CaRE-4- alloBMT program leverages significant in-kind funding and is built on a proven evidence-based model and the use of eHealth technology provides seamless cancer rehabilitation pre to post alloBMT. Moreover, this model has immense potential to be adapted and scaled to other centres. This trial will provide important new information on acceptability and effectiveness of CaRE-4-alloBMT with the goal to embed this model as part of standard of care for alloBMT patients. The inclusion of health economic lens allows our team to evaluate the program’s value for money to address sustainability.

**Appendix 1: Program Description and SUNDAE Framework**

**CaRE-4-alloBMT Program Description** **
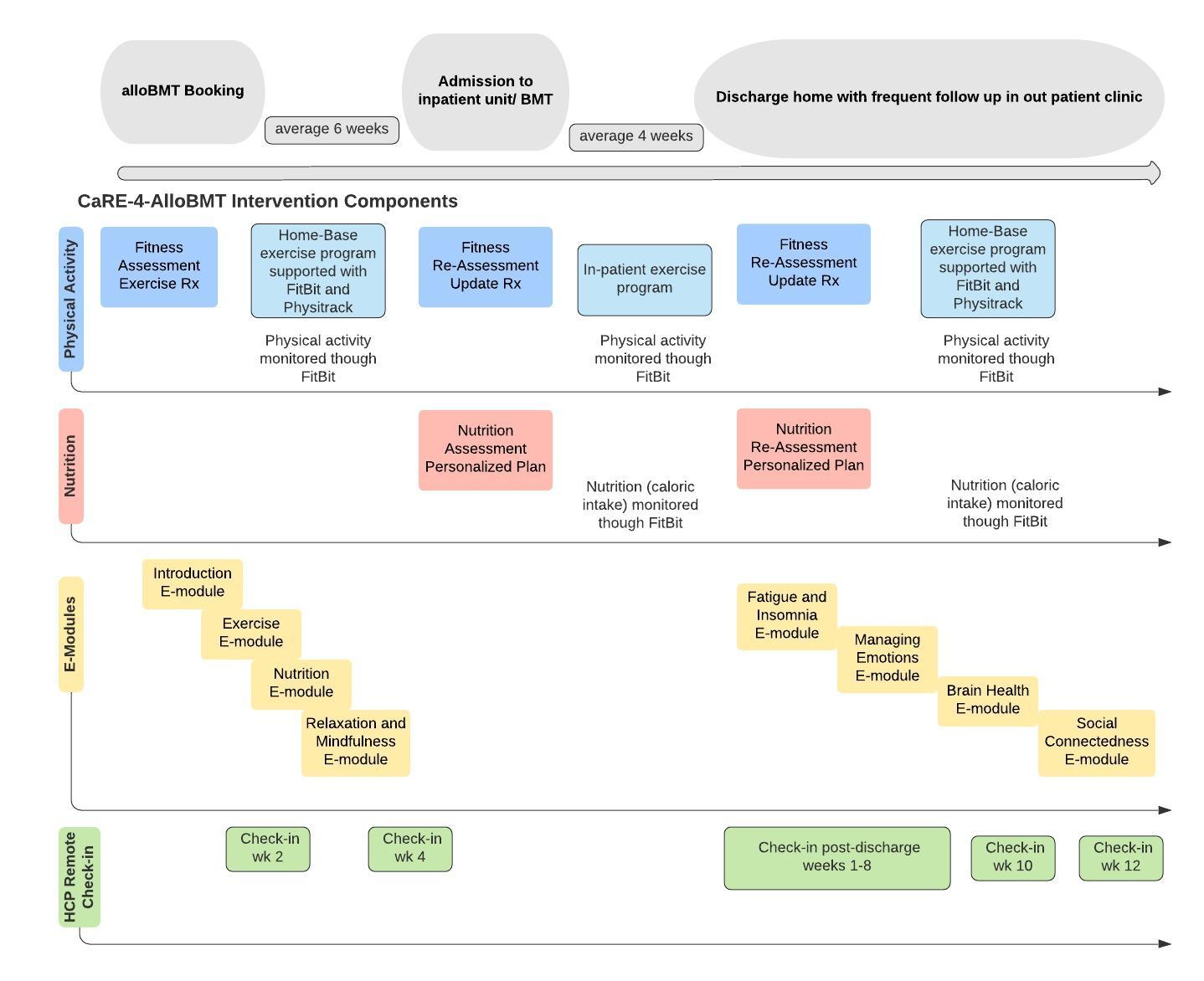
**


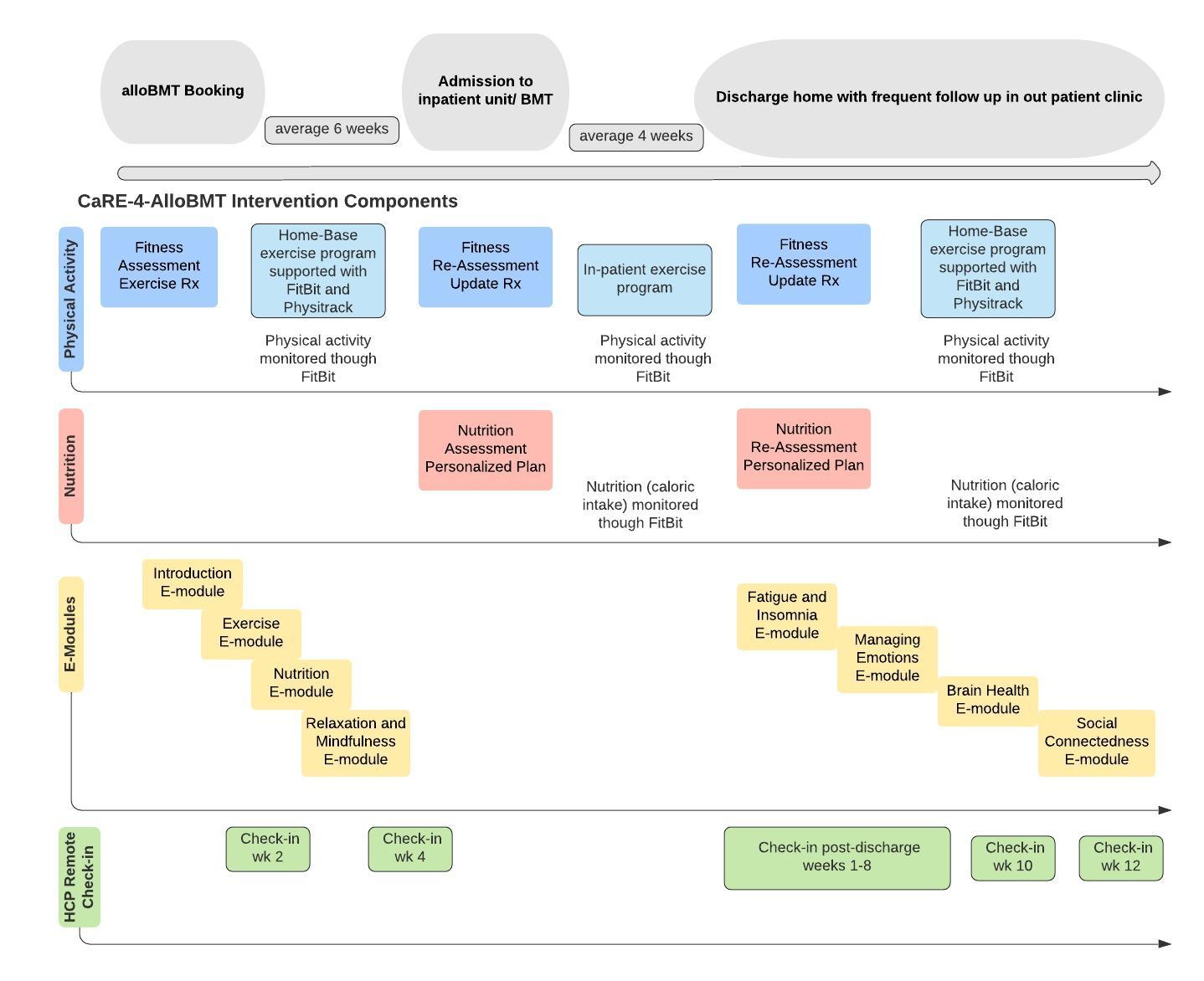


**SUNDAE Framework Elements in the CaRE program**


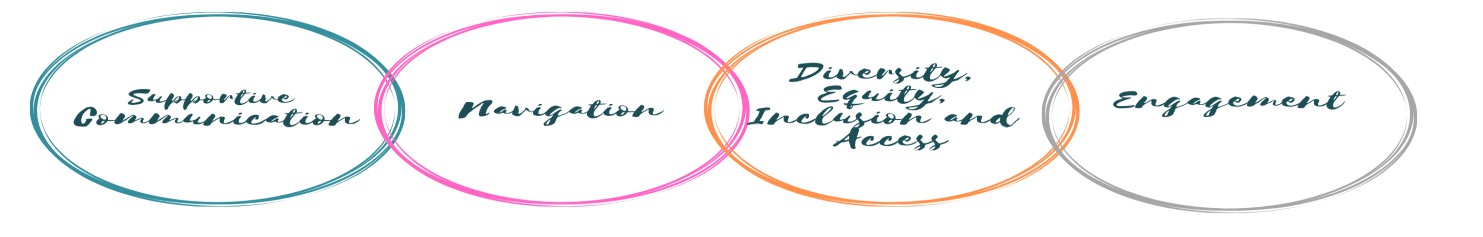

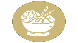


The CaRE program integrates principles of supportive communication including: fostering a therapeutic relationship, problem Defn, providing Info and support, responding to emotions & encouraging patients to take action.

The CaRE program includes proactive assessment and addresses gaps in care delivery. Further, its design focuses on reducing barriers and disparities in access to care & improving coordination, resulting in more efficient and effective supportive care.

eHealth, which is a large component of the CaRE program, can improve quality of care and access to care. Plus, it offers potential to increase the frequency and intensity of rehab

that patients are able to receive.

Within the CaRE program, engagement is viewed as a co- constructed *process* through the relationship with the alloBMT/CRS HCPs and as a *state* expressed through observable and supported behaviours.

**APPENDIX 2: Study Flow Chart and Timeline** **
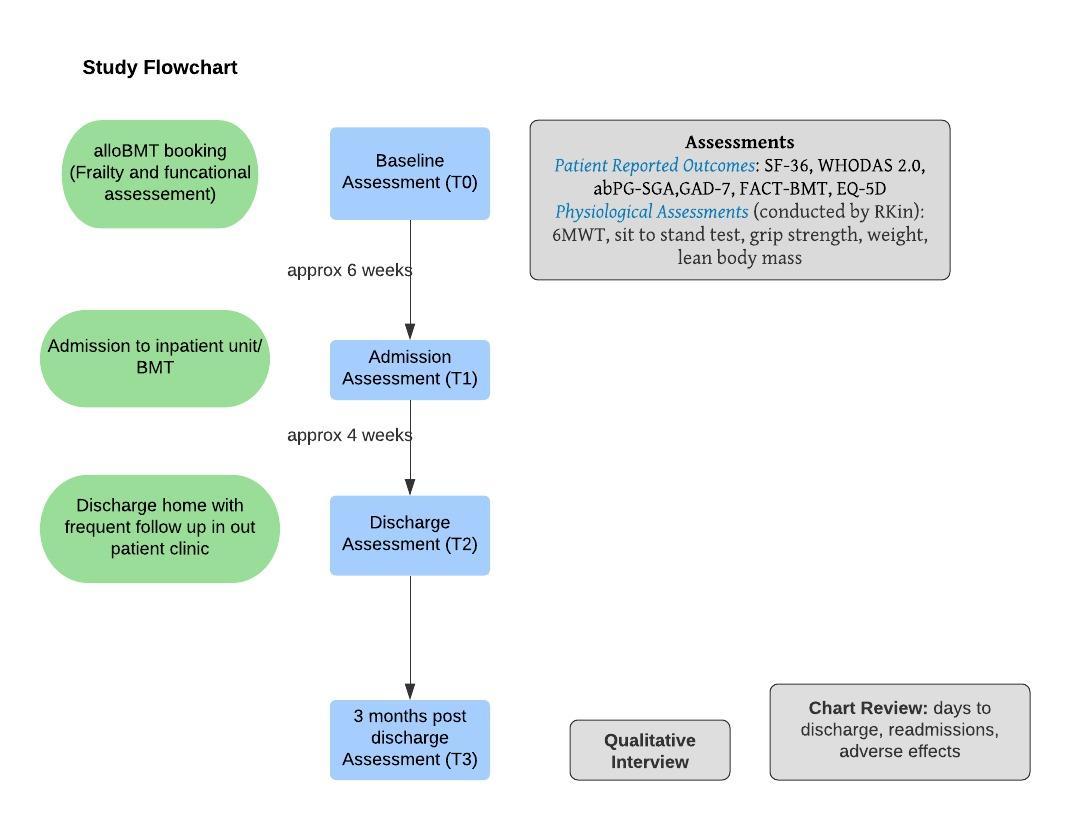
**


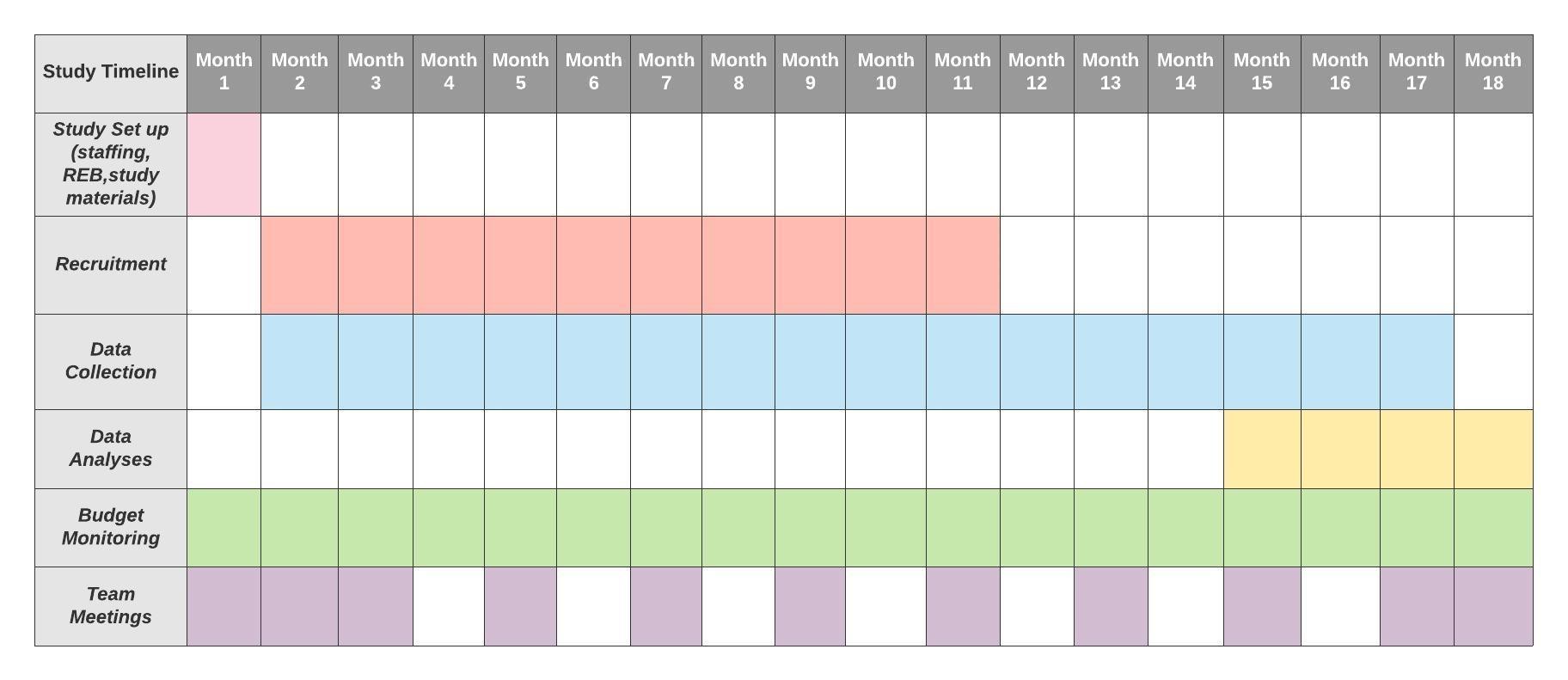


**Leveraged funds:** The CaRE-4-alloBMT program leverages existing resources and investments (CRS and alloBMT). This includes considerable resources which have been used to develop the existing CaRE program such as emodule development (~$92,000) and development of the clinician dashboard (~$20,000) and evaluation of the CaRE model (~$50,000). These funds have come from Foundation funds, Chair funds and grants (CCO, CCS).

**In-kind funding:** In-kind support is also provided for the current project through the Butterfield Drew Chair in Cancer Survivorship Research (Jones) and the Gloria and Seymour Epstein Chair in Cell Therapy and Transplantation (Mattsson). This incudes the development of an alloBMT nutrition specific emodule (in development ~$4,800) and Fitbits ($4,400). In addition, both the CRS and alloBMT team have already invested considerable staff time in the development of this program and are committed to continuing the support this program through staff resources.

**REQUESTED FUNDING**

**(I) SALARIES**

**1) Clinical Research Coordinator II (Masters level; 0.5 PPT)**

One (1) Clinical Research Coordinator II (CRC) will be hired for the duration of this study. This

individual will directly report to the PIs and their responsibilities will include: overall team coordination and supervision/support of staff; maintenance of a master study database; organization of study meetings; preparation of study reports. Further, this individual will also be responsible for financial activities related to the team, including; maintenance of accounting records; ensuring compliance with funding guidelines; preparation of all financial year-end reports and documentation; ongoing liaison with Research Financial Services. The budgeted hourly rate for the CRC II position is $37.00/hr as per University Health Network (UHN) Clinical Research Job Classification Matrix 2020 and Pay Scale for Clinical Research Coordinator I. Based 1,950 work hours per year (7.5 hr/day). The budget includes the standard

24% benefits package for full-time staff, as outlined by UHN, plus an estimated 3.0% annual cost of living/experience/merit increase per year, which is now standard for all UHN staff, including research personnel.

Year 1 Year 2 (6months) Subtotal

*Salary Rate $72,150 $74,315*

*Proportion of Year 1.0 0.5*

*FTE 0.5 0.5*

24% fringe benefits $8,658 $4,459

**Subtotal $44,733 $23,038 $67,771**

**2) Registered Kinesiologist (BA Level; 1.0 FTE)**

One (1) Registered Kinesiologist will be hired (0.4 = 2 days per week) to deliver care as well as

help to coordinate and support the study. The budgeted hourly rate for this position is $37.00/hr as per UHN Clinical Research Job Classification Matrix for registered kinesiologists. Based on

1,950 work hours per year (7.5 hr/day). The budget includes the standard required 24% fringe benefits package for contract employees, plus an estimated 3.0% annual cost of living/experience/merit increase per year.

Year 1 Year 2 (6 months) Subtotal

*Salary Rate $ 72,150* $74,315

*Proportion of Year 1.0* 0.5

*FTE*

*24% fringe benefits*

*0.4*

$6,926

*0.4*

$3,567

**Subtotal $35,786 $18,430 $54,216**

**3) Trainee Award**

One (1) Masters level graduate student will be hired for the duration of the 18 months. The

stipend will be budgeted at $28,403.74 a year.

Year 1 Year 2 (6 months) Subtotal

*Salary Rate* $28,403.74 $28,403.74

*Proportion of Year*

**Subtotal**

1.0

**$28,403.74**

0.5

**$14,201.87 $42,605.61**

**SALARIES TOTAL**

Year 1 Year 2 (6 months) Total

**Total $108,922.74 $55,669.87 $164,592.61**

**(II) EQUIPMENT**

**1. Computer Cost**

One laptop ($2000/unit) will be purchased to support the study.

Year 1 Year 2 (6 months) Subtotal

**Subtotal $2,000 $0 $2,000**

**2. Telephone Charges**

One RIS IP telephone will be rented to support this study for the duration of 18 months. The

cost to rent an RIS telephone is $35/month ($25 for phone rental + $10 for the phone line). The total cost for the duration of this study is $630 ($35x18).

Year 1 Year 2 (6 months) Subtotal

**Subtotal $420 $210 $630**

**EQUIPMENT TOTAL**

Year 1 Year 2 (6 months) Total

**Total $2,420 $210 $2,630**

**(III) SERVICES**

**1. Statistical Consultants – Biostatistical Support Unit, UHN**

The Biostatistics Support Unit at Princess Margaret provides research support at a rate of $65 per

hour. Support services include development and refinement of the data analysis strategy according to the study protocol, randomization, statistical analysis of data and analysis support as needed over the course of the study.

Year 1 Year 2 (6 months) Subtotal

*Number hrs 0 70*

**Subtotal $0 $4,550 $4,550**

**2. Health Economist**

A health economist (MSc/PhD level skillset with > 5 years working in economic evaluations using

person-level data) will be contracted from the CLEAR Health Economics Unit, Li Ka Shing Knowledge Institute, St. Michael’s Hospital. Under the supervision of study coinvestigator Dr. Wanrudee Isaranuwatchai, they will be responsible for facilitating and conducting an economic analysis, and supporting the interpretation, writing, and reporting of the results for this piece. The estimated numbers of hours for this work is 430 hours at a rate of $46.50. *See attached statement of work*.

Year 1 Year 2 (6 months) Subtotal

*Hourly rate Number of hours* **Subtotal**

**$0 $46.50**

**0 430**

**$0 $19,995 $19,995**

**3. RIS Network Charges**

The monthly network connectivity fees for the laptop purchased will be $38 ($25/month for the

device + $13 for the Microsoft licensing fee). The total cost for the duration of this study is $684 ($38x18).

Year 1 Year 2 (6 months) Subtotal

**Subtotal $456 $228 $684**

**SERVICES TOTAL**

Year 1 Year 2 (6 months) Total

**Total $456 $24,773 $25,229**

**(IV) KNOWLEDGE TRANSLATION and EXCHANGE (KTE)**

**1. Publication Fees**

Cost for 1 publication estimated at $2500 each

Year 1 Year 2 (6 months) Subtotal

*Cost n/a $*2,500

*Number 0 1*

**Subtotal $0 $2,500 $ 2,500**

**TOTAL AMOUNT REQUESTED**

Year 1 Year 2 (6 months) Subtotal

Salaries **$108,922.74 $55,669.87 $164,592.61**

Equipment **$2,420 $210 $2,630**

Services **$456 $24,773 $25,229**

KTE **$0 $2,500 $2,500**

**Total Requested $111,798.74 $83,152.87 $194,951.61**
